# 

**Published:** 1921-12

**Authors:** 


*Established 1886* *No. 1845, Vol. LXXI.*

*New Series.* Vol. I. No. 3 *December 1921* Price Sixpence

CONTENTS.

| | PAGE |
|---|---|
| NOTES AND COMMENTS | 61 |
| SMALLPOX AND VACCINATION<br>Dr. C. Killick Millard, M.D., D.Sc. | 65 |
| THE FUTURE OF THE HOSPITALS<br>Sir William J. Collins, K.C.V.O., M.S., M.D. | 68 |
| A PAGEANT FOR HOSPITALS | 70 |
| THE HEALTH OF LONDON | 71 |
| EDWARD JENNER—II. | 73 |
| A CHRISTMAS APPEAL FOR THE HOSPITALS | 77 |
| SMALLPOX AT NOTTINGHAM | 79 |
| REVIEW OF THE MONTH | 81 |
| FOLK-LORE IN MEDICINE | 82 |
| PASSING EVENTS AND TOPICS | 83 |
| REVIEWS OF BOOKS | 85 |
| OUR PRIZE LEAFLET | 90 |
| CORRESPONDENCE | 91 |

